# Kisspeptin Restores Pulsatile LH Secretion in Patients with Neurokinin B Signaling Deficiencies: Physiological, Pathophysiological and Therapeutic Implications

**DOI:** 10.1159/000336376

**Published:** 2012-02-24

**Authors:** Jacques Young, Jyothis T. George, Javier A. Tello, Bruno Francou, Jerome Bouligand, Anne Guiochon-Mantel, Sylvie Brailly-Tabard, Richard A. Anderson, Robert P. Millar

**Affiliations:** ^a^Faculté de Médecine Paris-Sud UMR-S693, Université Paris-Sud, France; ^b^INSERM U693, IFR93, Pharmacogénétique et Hormonologie, Assistance Publique-Hôpitaux de Paris, Hôpital Bicêtre, Le Kremlin-Bicêtre, France; ^c^Service d'Endocrinologie et des Maladies de la Reproduction, Pharmacogénétique et Hormonologie, Assistance Publique-Hôpitaux de Paris, Hôpital Bicêtre, Le Kremlin-Bicêtre, France; ^d^Service de Génétique Moléculaire, Pharmacogénétique et Hormonologie, Assistance Publique-Hôpitaux de Paris, Hôpital Bicêtre, Le Kremlin-Bicêtre, France; ^e^MRC Human Reproductive Sciences Unit, University of Edinburgh, Edinburgh, UK; ^f^Centre for Integrative Physiology, University of Edinburgh, School of Biomedical Sciences, Edinburgh, UK; ^g^Mammal Research Institute, University of Pretoria, Pretoria, and UCT/MRC Receptor Biology Unit, University of Cape Town, Cape Town, South Africa

**Keywords:** Deficient NKB signaling, GnRH pulse generation, Kisspeptin, Kisspeptin-10 infusion, Neurokinin B signaling deficiency, Pulsatile LH secretion, Hypogonadotropic hypogonadism

## Abstract

Pulsatile gonadotropin-releasing hormone (GnRH) is crucial to normal reproductive function and abnormalities in pulse frequency give rise to reproductive dysfunction. Kisspeptin and neurokinin B (NKB), neuropeptides secreted by the same neuronal population in the ventral hypothalamus, have emerged recently as critical central regulators of GnRH and thus gonadotropin secretion. Patients with mutations resulting in loss of signaling by either of these neuroendocrine peptides fail to advance through puberty but the mechanisms mediating this remain unresolved. We report here that continuous kisspeptin infusion restores gonadotropin pulsatility in patients with loss-of-function mutations in NKB (*TAC3*) or its receptor (*TAC3R*), indicating that kisspeptin on its own is sufficient to stimulate pulsatile GnRH secretion. Moreover, our findings suggest that NKB action is proximal to kisspeptin in the reproductive neuroendocrine cascade regulating GnRH secretion, and may act as an autocrine modulator of kisspeptin secretion. The ability of continuous kisspeptin infusion to induce pulsatile gonadotropin secretion further indicates that GnRH neurons are able to set up pulsatile secretion in the absence of pulsatile exogenous kisspeptin.

## Introduction

The seminal discovery of gonadotropin-releasing hormone (GnRH) [1] and subsequent studies have categorically established its role as the final neuroendocrine conduit for control of the gonadotropins, luteinizing hormone (LH) and follicle-stimulating hormone (FSH) by diverse central nervous system inputs [2,3]. LH and FSH act in concert to stimulate sex steroid secretion and gametogenesis in the testes and ovaries. Appropriate gonadotropin pulse frequency and amplitude is crucial for normal reproduction [2,4] and disruption is associated with pathological conditions such as hypothalamic amenorrhea (low pulse frequency) [5] and polycystic ovarian syndrome (high pulse frequency) [6]. However, the precise mechanisms whereby inputs such as metabolic status and sex steroids regulate GnRH secretion remained cryptic as GnRH neurons lack requisite receptors, estrogen receptor alpha [7] and leptin [8].

Recent discoveries of naturally occurring mutations have revolutionized our understanding of the neuroendocrine regulation of gonadotropins [2,9,10]. The discovery that mutations in the human and rodent G-protein-coupled receptor 54 (GPR54 also referred to as KISS1R) resulted in failure to progress through puberty and achieve adult reproductive function [11,12] led to the recognition that GPR54 and its cognate ligands, kisspeptins, are required for GnRH release and downstream gonadotropin secretion. The localization of kisspeptins to arcuate nucleus (ARC) neurons [9,10,13,14], and its potential involvement as a component of the GnRH pulse generator [9,13,14], suggested a role for kisspeptin in the regulation of GnRH pulse frequency. This postulate is supported by a slowing of LH pulse frequency after kisspeptin antagonist injection into the ARC of rats [15] and an increase in LH pulse frequency after kisspeptin administration in men [16]. Kisspeptin neurons express receptors for sex steroids which modulate kisspeptin gene expression, thereby providing a relay for steroid hormone feedback on GnRH neuron regulation [9,10,13,14].

Kisspeptin neurons in the ARC have been shown to also express neurokinin B (NKB) and dynorphin A (DYN) peptides [13,14,17,18] and are therefore called KNDY neurons [13]. Inactivating mutations in the genes encoding NKB (*TAC3*) and its cognate receptor, NK3R (*TACR3*), have also been recently shown, like GPR54 mutations, to result in hypogonadotropic hypogonadism; characterized by a failure to progress through puberty [19,20]. In contrast, *TACR3-*inactivating mutation in mice does not result in a phenotype of reproductive deficiency [21,22].

In order to contribute to an understanding of the hierarchy of roles of kisspeptin and NKB in the neuroendocrine control of GnRH pulsatility, we have administered kisspeptin to patients with hypogonadotropic hypogonadism resulting from naturally occurring loss-of-function mutations in the NKB ligand and its receptor. These patients are characterized by very low LH but normal or near-normal FSH circulating concentrations [19,20], consistent with low GnRH pulse frequency [23]. In contrast, inactivating mutations in the kisspeptin receptor result in low circulating concentrations of both LH and FSH [11,12]. Since GnRH neurons express the GPR54 receptor but apparently not NK3R in sheep and mice [18,24,25] and kisspeptin (KNDY) neurons express NK3R [26,27,28], we hypothesized that NKB secreted from KNDY neurons acts in an autocrine or paracrine manner to enhance kisspeptin secretion, and that kisspeptin alone is sufficient to elicit GnRH pulsatility. To test this postulate we infused kisspeptin at a GPR54- saturating concentration in patients with *TAC3-* and *TACR3-*inactivating mutations and demonstrated a restoration of LH pulsatility. This is the first indication of cooperative interactions of neuropeptides within a single neuronal population eliciting a pulsatile output essential for human health, and provides information on the hierarchy of kisspeptin and NKB in regulating GnRH secretion in humans.

## Methods

### Participants

Patients 1 and 2 were 21- and 31-year-old men harboring the c.209-1G_C homozygous mutation in the NKB gene (*TAC3*) [20]. This mutation is located in the IVS3 acceptor splice-site and unmasks a cryptic splice-site in IVS3, leading to the insertion of 22 nucleotides between exons 3 and 4 in the transcript. This insertion results in a frame-shift downstream of codon 67 and the emergence of a premature stop codon at position 76, causing the termination of translation upstream of the NKB coding region. Patient 3 was a 26-year-old woman with a homozygous deletion (c.483_499del) in the gene encoding NK3R (*TACR3*). This deletion leads to a frame-shift downstream of codon 161 resulting in a premature stop codon at position 183 truncating *TACR3* after the third transmembrane domain.

Patient 4 was a 28-year-old man harboring the homozygous c.738-1G_A mutation in the *TACR3* gene. This results in the loss of the natural splice acceptor site of intron 2 resulting in the truncation of the receptor after the second extracellular loop, thus eliminating transmembrane domains 5-7 and the associated intracellular and extracellular loops [20].

Testosterone enanthate therapy in the 3 male patients and estrogen-progestin replacement therapy in the female patient were discontinued at least 3 months before evaluation.

### Protocol

Kisspeptin-10 was custom-synthesized under GMP standards (Bachem GmbH, Weil am Rhein, Germany) and reconstituted in 5 ml sterile physiological saline as previously described [16]. The Paris Sud University and Bicêtre Hospital Ethics Committees approved the study and all participants gave their informed consent. Subjects were admitted to the Bicêtre Hospital at 08:00 h for 12 h of blood sampling every 10 min for 2 consecutive days as previously described [20]. Vehicle (NaCl 0.9%) or kisspeptin-10 (1.5 μg/kg/h was infused intravenously from 08:30 to 20:30 h on days 1 and 2, respectively). There were no adverse events related to kisspeptin-10 or vehicle infusions.

### Hormone Assays

Serum LH, FSH, inhibin B, testosterone and estradiol levels were measured by immunoassays as previously described [29,30,31,32]. The detection limits of the LH and FSH assays were 0.12 and 0.2 IU/l, respectively. The intra- and interassay coefficients of variation were 1.6 and 4.2% for LH and 2.7 and 5.5% for FSH, respectively.

### Statistical Evaluation of Gonadotropin Secretion

LH and FSH pulses were detected by the Thomas' algorithm, by analysis of LH and FSH concentrations in samples collected at 10-min intervals, as reported elsewhere [20,29,30]. Mean serum LH and FSH levels during saline (n = 72 measurements of each gonadotropin) or kisspeptin (n = 72 measurements of each gonadotropin) infusions were compared in each of the 4 patients by the paired parametric t test.

In the 3 male patients (subjects 1, 2 and 4; table 1) mean serum testosterone levels (n = 12 measurements during each treatment), mean serum inhibin B levels (n = 12 measurements during each treatment) and mean serum estradiol levels (n = 12) were compared by the paired parametric t test.

Statistical differences in mean pulse frequencies, hormone concentrations and area under the curve (AUC) were analyzed using GraphPad Prism 5 using a paired t test, where one-tailed p values <0.05 were considered statistically significant.

## Results

**Fig. 1.**
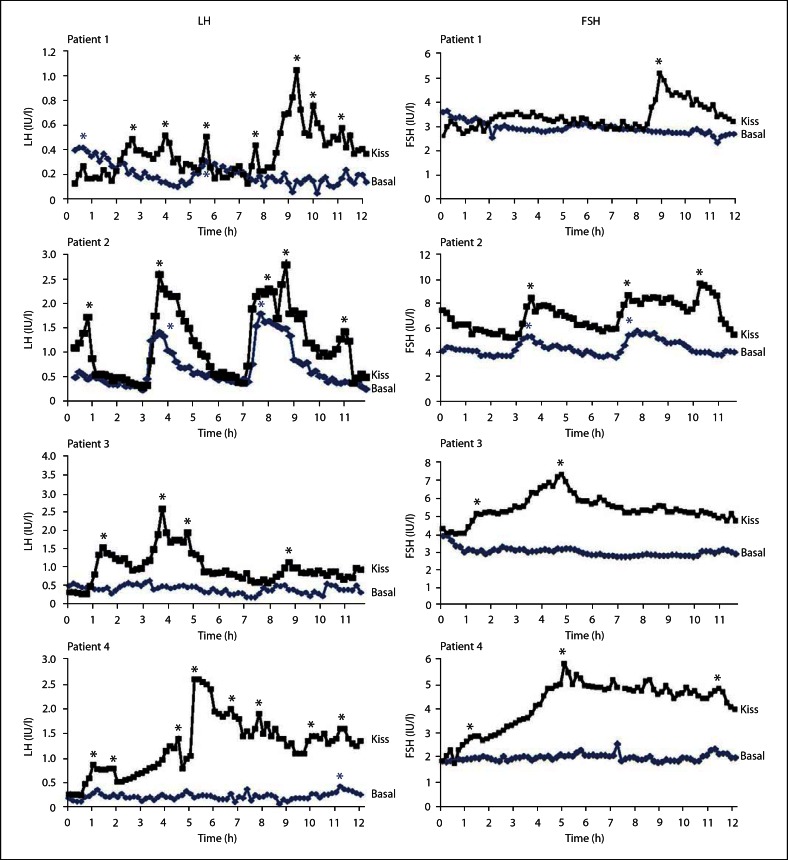
Secretory pattern of LH and FSH in 2 patients with NKB *(TAC3)* biallelic mutations (1 and 2) and 2 patients with NK3R *(TACR3)* biallelic mutations (3 and 4) during infusion of saline (▪) or kisspeptin-10 (▪). Asterisks denote LH pulses as identified with the Thomas' algorithm.

**Fig. 2.**
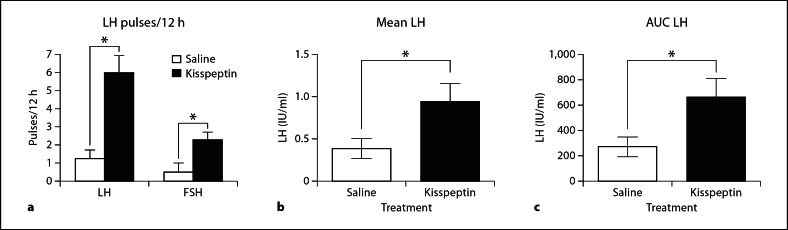
Effects of continuous kisspeptin-10 on LH pulse frequency, mean LH levels and AUC: (**a**) mean (±SEM) frequency of gonadotropin pulses, (**b**) mean (±SEM) LH levels, and (**c**) mean (±SEM) LH AUC in 4 patients with hypogonadotropic hypogonadism caused by NKB *(TAC3)* or NK3R *(TACR3)* mutations receiving vehicle (white column) and kisspeptin (black column). * p < 0.05 paired t test.

The 2 patients with *TAC3-*inactivating mutations and the 2 patients with *TACR3-*inactivating mutations were of reproductive age between 21 and 31 years and have been previously described in detail [20]. In this study we confirmed that they all had low circulating LH concentrations but normal or near-normal FSH, low inhibin B, low testosterone in the 3 males and low estradiol in the female patient (table 1). Circulating LH and FSH concentrations and pulsatility during 12 h vehicle (saline) or kisspeptin-10 (1.5 μg/kg/h) infusions are shown in figures 1 and 2 and in table 1. During the 12 h of vehicle infusion (10-min sampling frequency) LH concentrations were lower than normal in all 4 patients with either ligand or receptor-inactivating mutations (fig. 1, 2; table 1). Patient 2, who had the identical inactivating mutation to patient 1, had residual LH activity as there were two distinct LH pulses and a higher mean LH, albeit well below normal (fig. 1, 2; table 1).

Intravenous infusion of kisspeptin-10 elicited a slow initial LH response followed by a sustained reinstatement of LH pulses in all 4 patients and a concomitant increase in gonadal steroids and inhibin B (fig. 1, 2; table 1). There was also a significant increase in the mean LH concentration and LH AUC (fig. 2). Although FSH concentrations were normal on vehicle fusion, there was a significant stimulation in mean concentrations and pulse frequency (fig. 1, 2; table 1). Interestingly, quite a few of the FSH pulses were coincident with LH pulses, which is not readily discernible in normal subjects.

## Discussion

### Effects of Deficient NKB Signaling on Gonadotropins

The scenario of normal or near-normal FSH (2.1-5.4 IU/ml) but low LH (0.2-0.62 IU/ml) and gonadal steroid levels in the patients with NKB signaling mutations is compatible with deficient (low) GnRH pulse frequency, which favors FSH gene transcription [23] and secretion [33] over that of LH. This contrasts with inactivating mutations of kisspeptin signaling, which results in low circulating concentrations of both gonadotropins [2,11,12]. This also indicates that inactivating mutations of NKB signaling give rise to a less severe hypogonadotropic phenotype and suggests that NKB signaling is upstream of kisspeptin signaling in the neuroendocrine hierarchy of GnRH regulation.

Both patients with inactivating mutations in *TACR3* and patient 1 with an inactivating mutation in *TAC3* had very low LH levels (0.2, 0.22 and 0.42 IU/ml) and no evidence of pulses. Thus, inactivating mutations of either the ligand or receptor have a similar phenotype suggesting that compensatory utilization of a ligand or receptor homolog does not occur. However, there have been reports of spontaneous recovery of fertility in patients with mutations in NKB signaling [34] but this did not take place in our patients. Interestingly, patient 2, who had the same mutations of *TAC3* as patient 1 and which result in a failure to produce active proteins, had higher LH (0.65 IU/ml) and two pulses during the control 12 h infusion. This suggests that dependence of LH pulse generation on NKB input varies between individuals. Individual variations in severity of phenotype have also been noted in kisspeptin and kisspeptin receptor knockout mice [35].

### Responses to Kisspeptin-10 Infusion

Continuous kisspeptin infusion stimulated LH responses but these were generally slower and at a lower concentration than that observed in our previous studies utilizing the same protocol in normal men [16] and in other studies with acute administration of kisspeptin-10 [36] or kisspeptin-54 in man [37], or continuous infusion in sheep [38]. However, the fold stimulation in the patients (mean 3.1-fold) was similar to that found in normal men (3.2-fold) receiving the identical continuous infusion dose [16]. The slower response and lower absolute level of response is possibly due to atrophy of GnRH neurons and gonadotropes in the afflicted patients. In support of the latter, in previous studies on these patients, it was necessary to repeat GnRH stimulations (priming) to elicit a robust LH response [20]. Recovery of the gonadotrope with prolonged stimulation seems evident as the LH pulses increased in amplitude with increasing time of exposure to kisspeptin-10 (fig. 1).

The patients also exhibited an increase in FSH during kisspeptin infusion, indicating that, although FSH concentrations were normal, there is capacity for additional response with an increase in GnRH pulse frequency. The increase (mean 1.7-fold) was much lower than the LH increase and similar to that seen for FSH in normal males (mean 1.8-fold) [16].

There were also significant increases in inhibin B in all patients and in testosterone in the males and estradiol in the single female studied, reflecting the increases in LH and FSH.

In addition to stimulating mean LH concentrations, continuous kisspeptin infusion clearly elicited pulsatile secretion of LH in all 3 of the patients who had an absence of LH pulses, and increased the pulse frequency from 2 to 5 in patient 2 over the 12 h. While LH pulses were restored to a frequency approaching normality (mean of 0.5 per hour compared with 0.7 per hour in our studies on untreated normal men) the amplitude and LH AUC was well below normal. This suggests that GnRH function is restored more readily than gonadotrope function after kisspeptin infusion. It may have been instructive to determine whether a continuation of kisspeptin infusion would have completely restored LH pulse amplitude and whether GnRH priming would have normalized the LH response to continuous kisspeptin infusion. Such studies will have to be the subject of future research as they were not feasible here for ethical and logistical reasons.

We have previously reported that continuous infusion of kisspeptin-10 at the same dose (1.5 μg/kg/h) increased the LH pulse frequency in normal men [16]. Thus the current findings support this observation but are even more persuasive as the baseline pulse frequency was nil or very low (mean 0.1 per hour) and was increased at least fivefold to 0.5 per hour. In contrast, the pulse frequency in the normal men was increased from 0.7 to 1.0 per hour. Since LH pulses are well established to be coincident with, and dependent on, GnRH pulses [39,40], also see reviews [2,3,41], it is reasonable to interpret that continuous kisspeptin-10 infusion in the current study increases GnRH pulse frequency.

### Kisspeptin-10 in GnRH Pulse Generation

A number of studies point to a role for kisspeptin in GnRH pulsatility in support of our findings. Kisspeptin is localized to KNDY neurons in the ARC [13,14,17,28] which is associated with GnRH pulse generation [42,43], and kisspeptin stimulates GnRH neuron firing [44,45,46]. Kisspeptin has also been shown to stimulate GnRH secretion from hypothalamic explants [47], and in vivo in monkeys [48]. Perhaps the most convincing evidence for a direct role of ARC kisspeptin in pulse generation was the demonstration that administration of kisspeptin antagonist in the ARC of ovariectomized rats decreased LH pulse frequency [15]. Microdialysis studies in pubertal monkeys also demonstrated that intracerebroventricular kisspeptin antagonist inhibited GnRH pulses [46]. Since the multi-unit electrical activity recorded in the ARC is coincident with LH pulses [42,43] it is tempting to propose that multi-unit electrical activity in the ARC will be correlated with pulsatile kisspeptin secretion which in turn generates GnRH pulsatility [13,14,49]. However, our demonstration that continuous kisspeptin infusion increases LH pulse frequency in normal men and in our patients with NKB signaling deficiencies appears to run counter to this, and suggests that the delivery of kisspeptin to the GnRH neuron in a continuous modality is sufficient to entrain GnRH pulsatility, either independently or in concert with other neural inputs. The sustained stimulation of GnRH neuron firing on exposure to kisspeptin might be interpreted as supporting this suggestion as the stimulation of firing lasts about 20 min [44,45,46] which is much more protracted than a GnRH pulse. The above interpretation does not intend to imply that kisspeptin is not delivered to the GnRH neuron in a pulsatile modality [48] but simply that, while kisspeptin is a prerequisite for GnRH pulsatile secretion, pulsatile delivery is not, and GnRH pulsatility is apparently independent of kisspeptin pulsatility. There is evidence that GnRH neurons have the ability to independently generate pulsatile GnRH release [50,51] and kisspeptin input may reinforce this endogenous rhythm.

It can be argued that the restoration of LH pulsatility in our patients might arise through a low level of endogenous kisspeptin pulsatility superimposed on the constant circulating kisspeptin provided by continuous infusion. However, this is an unlikely explanation as the infusion dose we employed gives rise to near-maximum LH stimulation [16], thus negating any major contribution from endogenous kisspeptin. Moreover, constant infusion of kisspeptin in normal men with normal LH pulsatility was shown to increase LH pulse frequency [16].

### Hierarchy of NKB and Kisspeptin in GnRH Stimulation

The failure of humans with inactivating mutations in the kisspeptin/GPR54 or NKB/NK3R signaling systems to progress through puberty [11,12,19,20], and the restoration of gonadotropin secretion by exogenous pulsatile GnRH administration [2,20] indicates that these systems are proximal (upstream) to GnRH neurons. The ability to inhibit kisspeptin-stimulated gonadotropin release with GnRH antagonists and the localization of GPR54 to GnRH neurons further supports this conclusion [2,9,10,13,52].

**Fig. 3.**
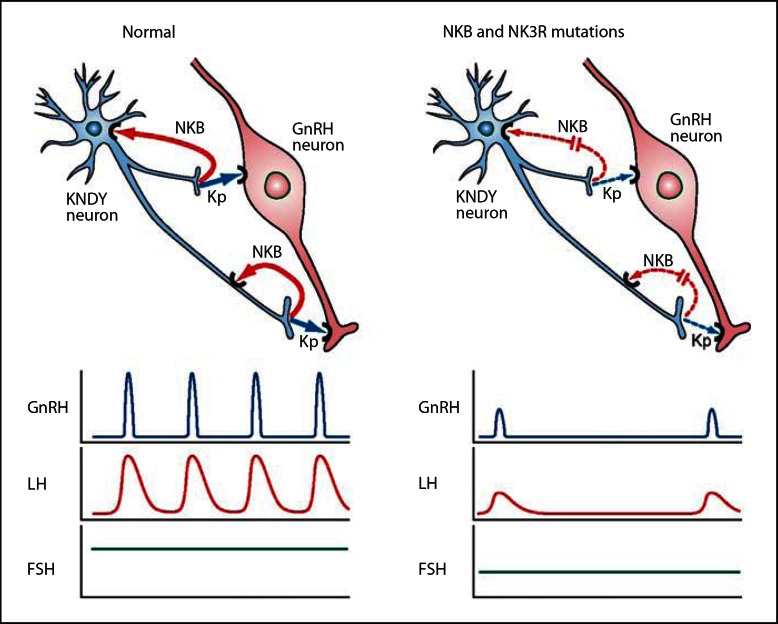
Schematic of proposed actions of a KNDY neuron on GnRH secretion summarizing findings from human and animal studies. Impacts of NKB and kisspeptin release on GnRH neuron secretion and LH and FSH responses in normal subjects (left) and patients with NKB- and NK3R-inactivating mutations (right). In normal subjects NKB acts in an autocrine (shown) or possibly paracrine (not shown) modality to reinforce kisspeptin secretion, which stimulates the GnRH neuron to secrete GnRH in pulses with a frequency interval of about 90 min. This results in corresponding LH pulses and normal FSH levels. In patients with NKB-inactivating *(TAC3)* and NK3R-inactivating *(TACR3)* mutations the absence of NKB stimulation of the KNDY neuron results in low kisspeptin secretion and resulting low GnRH pulse frequency with correspondingly low LH pulse frequency and amplitude, and lower end of normal FSH secretion. Continuous infusion of kisspeptin overrides this deficiency to restore the normal pattern of LH pulses and a small increase in FSH. Note that the most parsimonious scheme involving kisspeptin and NKB is presented. In reality a greater complexity of regulation of the KNDY neuron including DYN and other regulators, as well as additional inputs into the GnRH neuron will be operative.

While this proximal position of kisspeptin and NKB to GnRH in the neuroendocrine hierarchy is indisputable, the precise mechanism by which they interactively regulate GnRH pulsatility has not been established. An important outcome of our study is that kisspeptin alone in the absence of NKB signaling is sufficient to generate LH (and by inference GnRH) pulsatility, suggesting NKB actions are upstream of kisspeptin. Since NKB and kisspeptin are co-localized in the KNDY neurons in the ARC [13,14,17,18] (which plays a role in GnRH pulse generation) [42,43,49], the observation that continuous infusion of kisspeptin can restore LH pulsatility in our patients suggests that NKB has an autocrine/paracrine action to induce kisspeptin and thus GnRH secretion as schematically represented in figure 3. This is similar to schemes proposed by others [13,14,26,27,28,53].

Our deduction from the current findings that NKB operates upstream of GnRH neurons by stimulating kisspeptin secretion from KNDY neurons, and not by direct activation of GnRH neurons, is supported by some data from animal studies. Firstly, *TAC3R* is expressed in ARC KNDY neurons in mice [27], rat [54] and sheep [25] while *TAC3R* expression was reported to be absent, or was not observed, in GnRH neurons of mice and sheep [13,25]. Secondly, studies on the effects of NKB and analogs in mice, monkeys, sheep and goats on LH secretion have suggested a modulatory role for NKB in the release of kisspeptin by KNDY neurons in the ARC [13,14,24,27,28,49,55]. Thirdly administration of a NKB agonist to rats induced c-fos expression in KNDY neurons but was not observed in GnRH neurons in this study [28]. Fourthly, we have shown that kisspeptin antagonist alone slowed LH pulse frequency when administered into the ARC in ovariectomized adult rats [15] and apparently slowed LH pulses in ovariectomized sheep [46]. We have further supported the concept that NKB acts upstream of kisspeptin by demonstrating that the increase in LH pulse frequency resulting from treatment with the NKB agonist, senktide, in intact juvenile female rats is prevented by kisspeptin antagonist [P. Grachev et al., unpubl. data]. Although these findings present a persuasive argument, there have been reports of TAC3R expression in rat GnRH neurons [26,54] which apparently runs counter to our interpretation. These contradictory reports on expression of the NKB receptor on GnRH neurons require resolution through the demonstration of functional receptor protein or not in GnRH neurons. Interpretation of NKB's role in gonadotropin regulation is further complicated in rodents as it appears to have both inhibitory and stimulatory effects depending on physiological status [27,28,56] and NKB knockout mice do not apparently have impaired reproduction in contrast to our patients [21,22]. The findings in rodent models therefore appear to differ from sheep [24], monkeys [55] and now humans, which consistently demonstrate a stimulatory role for NKB upstream of kisspeptin.

### Therapeutic Implications

Our findings have implications for therapeutic interventions in various dysfunctions of the reproductive system. In conditions of decreased LH production and near-normal FSH secretion (i.e. due to low GnRH pulse frequency) such as hypothalamic amenorrhea or delayed puberty [5], restoration of LH and normal reproductive function may potentially be achieved by treatment with NKB or kisspeptin agonists. Unlike GnRH that needs to be administered in a pulsatile manner to elicit augmentation of LH pulse frequency, it appears that kisspeptin can be administered continuously for at least 3 days [George et al., unpubl. data] to stimulate LH and gonadal steroid hormone secretion. Conversely, conditions characterized by high LH pulse amplitude and frequency such as polycystic ovarian syndrome [6] may be treatable by NKB antagonists or kisspeptin antagonists. We have previously reported that kisspeptin antagonists reduce LH pulse frequency and amplitude but do not lower basal LH levels [15,46,57] in contrast to GnRH antagonist administration, suggesting that kisspeptin or NKB antagonists may be suitable for partial suppression of gonadotropins and gonadal steroids in hormone-dependent diseases, such as endometriosis and benign prostatic hyperplasia, without inducing bone loss or hot flushes that occur with GnRH analog therapy [57]. A potential limitation in the therapeutic use of kisspeptin agonists is tachyphylaxis, which has been reported with long-term (2 weeks) administration of kisspeptin-54 to women with hypothalamic amenorrhea [58] and rapidly in juvenile [59] and adult [53] monkeys. However, we have observed no evidence of tachyphylaxis in the current study and in men receiving continuous infusion of kisspeptin-10 at near-maximal LH-stimulating levels over 22 h [16].

In summary, our findings that kisspeptin administration restores LH pulsatility in infertile patients with NKB- or NK3R-inactivating mutations have provided insight into the hierarchy and interplay of kisspeptin and NKB in GnRH pulse generation in humans. The findings suggest a number of novel approaches to treating dysfunctions of the reproductive system and hormone-dependent diseases.

## Disclosure Statement

The authors have no conflicts of interest to disclose.

## Figures and Tables

**Table 1:** Mean (±SD) serum LH, FSH, inhibin B, testosterone and estradiol concentrations in patients with congenital hypogonado-tropic hypogonadism caused by NKB (patients 1 and 2) or NK3R (patients 2 and 3) biallelic mutations receiving vehicle (saline) and then kisspeptin-10 (Kp10) infusion

	Patient 1 (male)		Patient 2 (male)		Patient 3 (female)		Patient 4 (male)		Normal range^1^
	saline	Kp10	saline	Kp10	saline	Kp10	saline	Kp10	
LH, IU/l	0.20±0.1	0.40 ± 0.2^***^	0.62 ± 0.41	1.21 ± 0.60^***^	0.22 ± 0.06	1.25 ± 0.56^***^	0.42 ± 0.10	1.12 ± 0.43^***^	M: 2.6–6.5; F: 4–7.0
FSH, IU/l	2.9 ± 0.2	3.4± 0.5^***^	4.4 ± 0.6	7.1 ± 1.1 ^***^	2.1 ± 0.1	4.2 ± 0.9^***^	3.0 ± 0.2	5.4 ± 0.7^***^	M: 2.7–7.4; F: 4–7.4
Inhibin B									
pg/ml	15.9 ± 3.2	27.4 ± 4.6^*^	10.4 ± 1.6	19.1 ± 3.1 ^*^	8.15 ± 2.9	26.2 ± 4.7^**^	66 ± 7.4	112 ± 13.7^**^	M: 80–330; F: 60–125
Testosterone									
ng/ml	0.13±0.1	0.4 ± 0.2^*^	0.12 ± 0.08	0.6 ± 0.14^*^	ND	ND	0.42 ± 0.10	1.12 ± 0.43^*^	M: 3.5–±.5
Estradiol									
pg/ml	ND	ND	ND	ND	6.4 ± 1.9	18.2 ± 3.1^**^	ND	ND	F: 25–90

During each treatment, 12 random measurements of inhibin B, testosterone and estradiol were performed. ND = Not determined.

* p < 0.05;

** p < 0.01;

*** p < 0.0001 (paired t test).

1 Normal hormonal range in postpubertal males (M) and females (F).
